# A Large Hyperkeratotic Plantar Lesion: An Atypical Presentation of Acquired Digital Fibrokeratoma

**DOI:** 10.7759/cureus.105593

**Published:** 2026-03-21

**Authors:** Sri Naidnur, Eric Sandrock, Kelly Maedo, Emily DeSantis, Rick Lin

**Affiliations:** 1 Dermatology, Oasis Dermatology Group, McAllen, USA; 2 Dermatology, HCA Healthcare Corpus Christi Medical Center-Bay Area Program, McAllen, USA; 3 Dermatopathology, Sagis Diagnostics, Houston, USA

**Keywords:** acquired acral fibrokeratoma, acquired digital fibrokeratoma, adfk, atypical, hyperkeratotic, pedunculate, plantar

## Abstract

Acquired digital fibrokeratoma (ADFK) is a benign fibrous proliferation that typically presents as a small, solitary papule on a digit, often surrounded by a characteristic collarette of scale. Giant variants are uncommon and may pose diagnostic challenges, particularly when arising in atypical locations. We report a 56-year-old man with a 14-year history of a progressively enlarging hyperkeratotic lesion on the plantar surface of the foot. Excisional biopsy revealed a lesion measuring 4.4 × 2.9 × 1.5 cm, and histopathologic evaluation confirmed the diagnosis of ADFK. This case highlights that ADFK may present as a large plantar lesion with atypical clinical features and underscores the importance of histopathologic evaluation in morphologically unusual acral tumors.

## Introduction

Acquired digital fibrokeratoma (ADFK), also termed acquired acral fibrokeratoma when occurring on non-digital acral sites, is a benign fibrous tumor most commonly arising on the fingers and toes [1]. These lesions typically occur in middle-aged adults and present clinically as a solitary, skin-colored to pink, dome-shaped papule or finger-like projection often surrounded by a characteristic collarette of scale at its base [1,2]. While the etiology remains largely idiopathic, chronic mechanical irritation or trauma has been suggested as a potential provocative factor [1].

Most lesions measure less than 1 cm in diameter [1,2]. Lesions exceeding approximately 1-1.5 cm are categorized as “giant” variants and are uncommon [2,3]. Giant ADFKs occurring on weight-bearing surfaces, such as the plantar foot, may lack classic clinical features and can broaden the clinical differential diagnosis. In a retrospective series of 124 patients, lesions greater than 1 cm accounted for 7% of cases (9/124), and non-digital locations comprised approximately 24% of reported lesions; recurrence following excision occurred in 4% of cases (5/124) [3]. Complete surgical excision is considered curative in most cases [4].

We report a giant hyperkeratotic plantar ADFK with an unusual clinical presentation, highlighting the diagnostic challenges of large acral lesions.

## Case presentation

A 56-year-old man presented with a 14-year history of a progressively enlarging lesion that began as a small papule on the left plantar foot. He denied pain, bleeding, ulceration, rapid changes in morphology, or preceding trauma. Despite the lesion’s substantial size, he reported no functional impairment and was able to wear standard footwear without difficulty. The patient had presumed the lesion to be a plantar wart for several years and had not previously sought medical evaluation.

**Figure 1:**
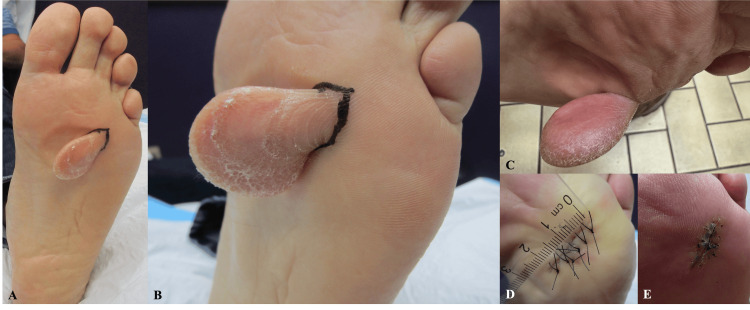
Clinical images. A. Clinical photograph demonstrating a solitary, markedly hyperkeratotic, pedunculated exophytic lesion on the plantar surface; the surrounding skin is marked to outline planned surgical margins. B. Closer view demonstrating preserved proximal dermatoglyphic lines with prominent distal hyperkeratosis and scale. C. Medial view highlighting the elongated exophytic morphology and indentation of the adjacent plantar skin from chronic pressure. D. Immediate post-excisional image demonstrating sutured closure; the surgical defect measured approximately 2.2 cm. E. Three-week follow-up demonstrating the surgical site after suture removal.

Physical examination revealed a solitary, symmetric, well-circumscribed, markedly hyperkeratotic, pedunculated exophytic mass on the anterolateral eminence of the left plantar foot (Figures 1A, 1B), measuring approximately 4.0 × 2.8 cm clinically, on a weight-bearing surface, with features suggestive of a long-standing process. Dermatoglyphic lines were preserved at the proximal base, while prominent hyperkeratosis and thick scale were present distally. A subtle indentation of the adjacent plantar skin was observed, consistent with chronic pressure from the lesion (Figure 1C). A classic collarette of scale encircling the base was not observed.

The clinical differential diagnosis included exophytic plantar callus, verruca plantaris, epithelioma cuniculatum plantare, and superficial acral fibromyxoma. Given the lesion’s size and exophytic morphology and patient preference, excisional biopsy was performed for definitive diagnosis.

Gross examination of the excised specimen demonstrated a firm mass measuring 4.4 × 2.9 × 1.5 cm. The surgical defect was repaired with linear closure (Figure 1D). Sutures were removed three weeks postoperatively without complications (Figure 1E). At follow-up, the surgical site was well healed, and the patient remained asymptomatic with no evidence of recurrence.

**Figure 2:**
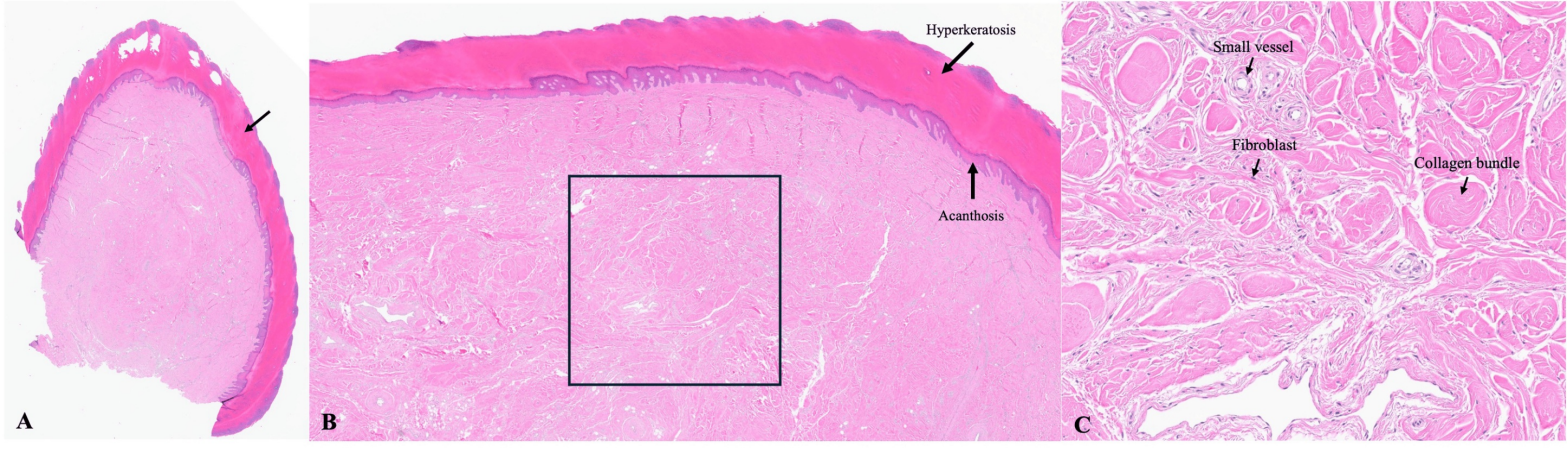
Histopathologic findings (H&E stain). A. Low-power view at 1× magnification demonstrating a dome-shaped lesion; the arrow indicates the hyperkeratotic epidermis overlying a fibrous dermal core. B. Intermediate view at 2× magnification showing epidermal hyperkeratosis and acanthosis. The boxed area highlights the dermal core shown at higher magnification in panel C. C. Higher-power view at 10× magnification of the dermal core demonstrating thickened collagen bundles, small blood vessels, and plump fibroblasts (marked with arrows).

Histopathologic examination on hematoxylin and eosin staining revealed a hyperkeratotic and acanthotic epidermis overlying a dermal proliferation composed of plump fibroblasts, small blood vessels, and thickened collagen bundles arranged in a haphazard array (Figures 2A-2C). These findings confirmed the diagnosis of ADFK. Given its non-digital plantar location, the lesion was more precisely classified as an acquired acral fibrokeratoma.

## Discussion

Giant variants of ADFK are uncommon. A focused literature search using the terms “giant acquired digital fibrokeratoma” and “acquired acral fibrokeratoma” was performed, and reference lists of relevant articles were reviewed to identify additional reports; selected cases are tabulated in Table 1 for comparison [4-10].

**Table 1:** Selected reported cases of giant acquired digital fibrokeratoma (≥3 cm), highlighting the lesion size and anatomic location for comparison with the present case (bolded).

Dimensions (cm)	Location	Age/Sex	Author (Year)
4.4 × 2.9 × 1.5	Left plantar foot	56/M	Present case (2026)
4.0 × 2.5 × 2.1	Left thumb	51/M	Bulam et al., 2013 [5]
4.0 × 1.5	Left foot	15/M	Frydman et al., 2010 [6]
3.8 × 3.2 × 1.5	Right great toe	33/F	Kakurai et al., 2003 [4]
3.5 × 1.5	Left index finger	50/M	Inamadar et al., 2013 [7]
3.0 × 2.2 × 1.0	Right heel	50/M	de Freitas et al., 2008 [8]
3.0 × 1.6 × 1.2	Left hallux	48/M	Ali et al., 2015 [9]
3.0	Fifth toe	50/M	Fujihara et al., 2025 [10]

Cases summarized in Table 1 [4-10] demonstrate that giant ADFKs most commonly arise on the digits and tend to occur in middle-aged adults, with a predominance in men. Reported lesions measure approximately 3-4 cm in greatest dimension, placing the present case (4.4 cm on gross examination) at the upper end of the reported size spectrum. Although digital involvement predominates, a smaller number have been reported at non-digital acral locations, including the foot and heel [6,8]. Table 1 was constructed by selecting reports describing “giant” ADFKs with dimensions similar to those of the present case to facilitate size comparison. It should be noted that prior reports do not consistently specify whether measurements represent clinical dimensions or gross specimen size, which limits precise comparison across cases. Nevertheless, recognition of these larger variants is important, as lesions at the upper end of the size spectrum, particularly in non-digital locations, may mimic verrucous or neoplastic processes and increase the risk of misdiagnosis.

In this case, the lesion’s hyperkeratosis and location on a pressure-bearing plantar surface broadened the clinical differential diagnosis. An exophytic plantar callus was considered, given its association with mechanical stress. Verruca plantaris was also considered, particularly given the patient’s longstanding assumption that the lesion represented a wart; however, preservation of dermatoglyphic lines and absence of punctate thrombosed capillaries lowered suspicion, as plantar warts typically disrupt normal skin markings [1]. The lesion’s size and exophytic morphology also prompted consideration of epithelioma cuniculatum plantare, as well as superficial acral fibromyxoma.

Histopathologically, superficial acral fibromyxoma shows a myxoid stroma with CD34 positivity and storiform spindle cells, whereas exophytic plantar callus shows compact hyperkeratosis without a fibrous core, verruca plantaris shows papillomatosis with koilocytosis and thrombosed capillaries, and epithelioma cuniculatum shows endophytic growth with keratin-filled sinus tracts and minimal cytologic atypia, in contrast to the dense collagen bundles and fibroblastic proliferation characteristic of ADFK [1]. Accordingly, histopathologic evaluation is essential for definitive diagnosis and distinction from clinically similar acral tumors.

An additional notable feature was the absence of functional impairment. Despite measuring over 4 cm on a weight-bearing surface, the patient reported no gait abnormalities and was able to wear standard footwear. A shallow impression on the adjacent plantar skin suggested redistribution of pressure rather than focal weight bearing, likely related to the pedunculated architecture of the lesion.

## Conclusions

ADFK may present as an unusually large lesion in non-digital acral locations and may lack the classic features of smaller digital lesions, with a risk of misdiagnosis in these atypical presentations. Histopathologic evaluation confirmed a benign fibrous proliferation consistent with ADFK and remains essential for definitive diagnosis. This case provides a striking visual example of the impressive size and morphology that giant ADFK may exhibit on weight-bearing surfaces, highlighting the variable clinical presentations of this entity.
